# Efficacy and Safety of Hopantenic Acid in Pediatric Neurological and Developmental Disorders: A Systematic Review and Meta-Analysis

**DOI:** 10.7759/cureus.112111

**Published:** 2026-07-05

**Authors:** Prashant R Uttage, Demudu Babu Boddu, Subrat K Majhi, Davidson Devasia, Sudip Saha, Shikhar Patanjali, Suneel Godbole, Sunil K Agarwalla, Akanksha Sharma

**Affiliations:** 1 Department of Pediatrics Neurology, Utage Child Development Center, Hyderabad, IND; 2 Department of Neurology, Caring Hands Neuro Centre, Visakhapatnam, IND; 3 Department of Developmental Pediatrics, Apollo Hospitals, Bhubaneswar, IND; 4 Department of Pediatric Neurology and Epilepsy, Aster Medcity, Kochi, IND; 5 Department of Pediatrics, Chittaranjan Seva Sadan College of Obstetrics, Gynecology and Child Health, Kolkata, IND; 6 Department of Developmental Neurology, Neuroharmoni, Delhi, IND; 7 Department of Developmental Pediatrics, Chiranjeev Child and Development Center, Mumbai, IND; 8 Department of Pediatrics, Srirama Chandra Bhanja (SCB) Medical College and Hospital, Cuttack, IND; 9 Department of Psychiatry, Dr. Ram Manohar Lohia Institute of Medical Sciences, Lucknow, IND

**Keywords:** adhd, cerebral palsy, cognition, epilepsy, gaba-b receptor, hopantenic acid, language development, meta-analysis, neurodevelopmental disorders, pediatric

## Abstract

Neurodevelopmental disorders (NDDs) are pediatric conditions characterized by impairments in cognition, behavior, language, motor coordination, and social functioning. Hopantenic acid (N-pantoyl-γ-aminobutyric acid {GABA}), a selective partial GABA-B receptor agonist with neuroprotective and nootropic properties, has been utilized in various pediatric neurological disorders; however, consolidated evidence regarding its efficacy and safety remains limited. This systematic review and meta-analysis aimed to evaluate the therapeutic efficacy and safety of hopantenic acid in pediatric NDDs and to assess its impact across major neurodevelopmental domains represented by the language, cognition, motor coordination, and social communication (LCMS) framework. The review was conducted in accordance with Preferred Reporting Items for Systematic Reviews and Meta-Analyses (PRISMA) 2020 guidelines. Electronic databases, including PubMed/MEDLINE, SpringerLink, ScienceDirect, and regional neurological journals, were systematically searched for controlled clinical trials involving children and adolescents treated with hopantenic acid. Quantitative synthesis was performed using a random-effects Mantel-Haenszel model to calculate pooled risk ratios (RR) with 95% confidence intervals (CI), while outcomes unsuitable for pooling were synthesized narratively. Six controlled studies involving 439 pediatric participants were included, of which four studies (n=272) were eligible for meta-analysis. Pooled analysis demonstrated greater clinical improvement with hopantenic acid compared with control therapy (RR=2.13, 95% CI: 1.11-4.08; I²=81%; p=0.02). More pronounced effects were observed in epilepsy and anxiety-related conditions, while moderate benefits were reported in hypoxic-ischemic encephalopathy. Narrative synthesis further indicated improvements across LCMS-related domains, including speech and hearing development, attention, memory, psychomotor performance, behavioral regulation, visual-motor coordination, social functioning, emotional stability, learning ability, and overall quality of life, without increasing seizure frequency. Safety outcomes were favorable, with adverse events comparable to placebo and no treatment-related serious adverse events reported. Overall, current evidence suggests that hopantenic acid may provide potential clinical and neurodevelopmental improvement across multiple pediatric NDDs, with benefits extending beyond symptomatic relief to broader developmental functioning. Its multimodal neuroprotective and GABA-B modulatory mechanisms provide a plausible biological basis for these findings. Nevertheless, inter-study heterogeneity and limited geographic representation highlight the need for larger, well-designed randomized controlled trials with standardized neurodevelopmental endpoints.

## Introduction and background

Neurodevelopmental disorders (NDDs) constitute a diverse group of conditions that originate during the developmental period and are characterized by deficits in personal, social, academic, or occupational functioning across domains of motor skills, cognition, language, and social behavior [1]. The Diagnostic and Statistical Manual of Mental Disorders, Fifth Edition (DSM-5), classifies NDDs to include intellectual disability, communication disorders, autism spectrum disorder (ASD), attention deficit hyperactivity disorder (ADHD), specific learning disorders, and motor disorders, including tic disorders [2]. Although these conditions represent the core DSM-5 neurodevelopmental disorders, a number of pediatric neurological conditions, including epilepsy and hypoxic-ischemic brain injury, may also result in significant developmental, cognitive, behavioral, and functional impairments that overlap with neurodevelopmental outcomes. Developmental disabilities represent a substantial global burden, affecting approximately 317 million children worldwide, including an estimated 52.9 million children under five years of age [3]; in India, nearly one in eight children is affected by at least one neurodevelopmental disorder, with approximately 23 million children living with disabilities, many of whom remain undiagnosed and untreated [4]. The socioeconomic burden is considerable, with long-term implications for educational attainment, employment, and quality of life [5].

At the neurobiological level, the pathophysiology of NDDs is increasingly understood to involve disruptions in synaptic plasticity, particularly long-term potentiation (LTP), the cellular mechanism underlying learning and memory formation [6]. LTP, first described by Bliss and Lømo in 1973, represents a persistent strengthening of synaptic connections following repeated stimulation [7]. Disruptions in LTP, particularly those mediated by presynaptic γ-aminobutyric acid (GABA)-B receptor dysregulation, have been implicated in the cognitive and behavioral impairments observed across multiple NDD phenotypes [8]. Specifically, GABA-B autoreceptors at the presynaptic terminal modulate neurotransmitter release through neural oscillations and synchronization; their dysregulation disturbs the excitation-inhibition balance critical for normal neurodevelopment [9]. While these neurobiological mechanisms provide a plausible rationale for therapeutic investigation, mechanistic plausibility alone should not be considered evidence of clinical efficacy, which must ultimately be established through well-designed clinical studies.

NDDs involve impairments across multiple developmental domains, including language, cognition, motor coordination, social interaction, and adaptive functioning [1]. Therefore, assessment of therapeutic interventions requires a multidimensional developmental perspective beyond isolated symptom improvement. The language, cognition, motor coordination, and social communication (LCMS) framework was applied by the review authors as an exploratory narrative framework to organize multidimensional neurodevelopmental outcomes reported across studies [10]. The LCMS framework was developed for the purpose of evidence synthesis in this review and was not a prespecified framework used in the original clinical trials.

Hopantenic acid (N-pantoyl-GABA; also known as homopantothenic acid or calcium hopantenate) is a synthetic homolog of pantothenic acid (vitamin B5) in which the β-alanine moiety is replaced by γ-aminobutyric acid (GABA) [11]. This structural modification confers several pharmacological advantages as follows: unlike γ-aminobutyric acid (GABA), hopantenic acid readily crosses the blood-brain barrier, enabling direct central nervous system activity [12]. Hopantenic acid is a structural analog of GABA that modulates GABAergic neurotransmission through interactions with GABA receptor systems, particularly GABAB_BB​ receptors. Experimental and pharmacological studies suggest that its neuroprotective, anticonvulsant, and nootropic effects are mediated through modulation of GABAergic signaling while maintaining a favorable tolerability profile without marked sedative effects typically associated with classical GABAergic agonists [13]. Additionally, hopantenic acid stimulates anabolic processes in neurons, increases brain resistance to hypoxia and toxic substances, enhances glucose utilization in the cerebral cortex, and induces acetylcholine synthesis [14]. This multimodal pharmacological profile provides a theoretical basis for investigating hopantenic acid in neurodevelopmental and neurological disorders; however, its clinical utility must ultimately be established through robust clinical evidence [15].

Despite over four decades of clinical use in the Russian Federation (marketed as Pantogam and Pantocalcin) and historical use in Japan, the clinical evidence for hopantenic acid in pediatric NDDs has not been systematically consolidated in a meta-analytic framework [16]. The present review aimed to address this gap by synthesizing the available controlled clinical trial evidence on the efficacy and safety of hopantenic acid in pediatric neurodevelopmental disorders and selected neurological conditions associated with developmental impairment.

## Review

Methods

Study Design

**Figure 1:**
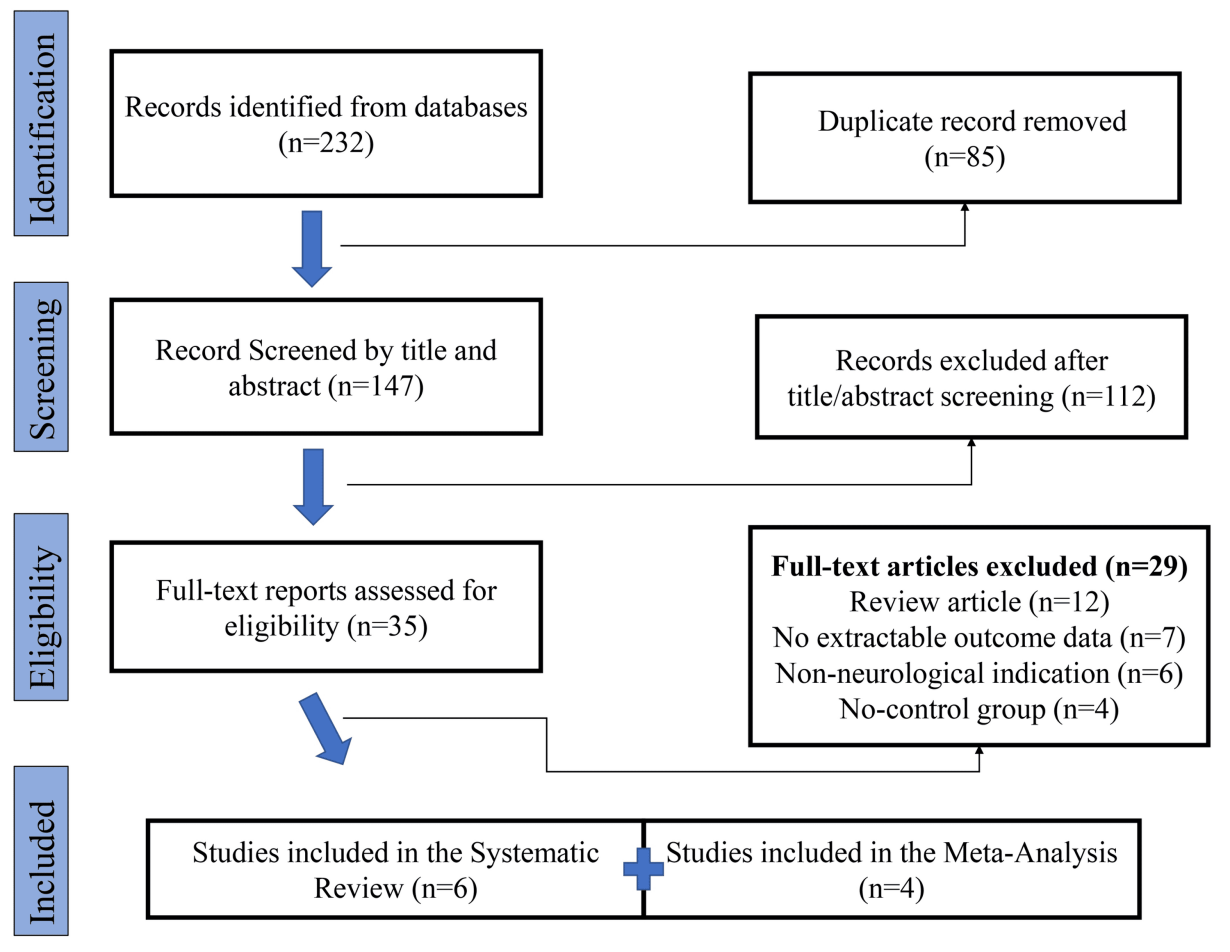
PRISMA 2020 flow diagram of study selection. PRISMA: Preferred Reporting Items for Systematic Reviews and Meta-Analyses

This systematic review with quantitative meta-analysis of dichotomous outcomes was conducted in accordance with the Preferred Reporting Items for Systematic Reviews and Meta-Analyses (PRISMA) 2020 guidelines (Figure 1). Given the heterogeneity of outcome measures and clinical populations across included studies, for studies reporting dichotomous outcomes (clinical improvement/responder rate), a quantitative meta-analysis was performed using Review Manager (RevMan 5.4.1; London, UK: The Cochrane Collaboration). Risk ratios (RR) with 95% confidence intervals (CI) were calculated using the Mantel-Haenszel method under a random-effects model. Due to heterogeneity in outcome definitions and study populations, only studies with extractable binary outcome data were included in the meta-analysis, while others were synthesized narratively.

Eligibility Criteria

Studies were included if they met the following criteria: (a) controlled clinical trials (randomized controlled trials {RCTs}, double-blind placebo-controlled trials, or comparative trials with a defined control group); (b) involved pediatric or adolescent patients with neurodevelopmental or neurological disorders. In addition, studies involving mixed adolescent and adult populations were considered when the investigated condition was relevant to neurodevelopmental or developmental outcomes and sufficient outcome data were available for analysis. Eligible studies also included those with (c) intervention consisting of hopantenic acid or its clinically marketed formulations (including calcium hopantenate, Pantogam, Pantocalcin, and Pantogam Active {D,L-hopantenic acid}). These formulations were considered eligible because they are structurally related hopantenic acid derivatives that share a common pharmacological mechanism based on GABAergic modulation, although differences between formulations were considered during interpretation of the findings; and (d) reported clinical outcomes related to cognitive, behavioral, or neurological improvement.

Exclusion criteria comprised uncontrolled case series, studies investigating D,L-hopantenic acid (Pantogam) exclusively in non-NDD or neurological indications, and studies lacking extractable outcome data. For quantitative synthesis (meta-analysis), only studies reporting outcomes that could be converted into dichotomous data (i.e., responders versus non-responders) in both intervention and control groups were included. Studies that reported outcomes as continuous variables, multidomain cognitive assessments, or lacked extractable responder data were included in the systematic review but analyzed narratively.

Information Sources and Search Strategy

A systematic literature search was conducted from database inception until January 31, 2026, in PubMed/MEDLINE, ScienceDirect, SpringerLink, and relevant regional journal sources, the Russian-language S.S. Korsakov Journal of Neurology and Psychiatry (Zhurnal Nevrologii i Psikhiatrii imeni S.S. Korsakova), Neuroscience and Behavioral Physiology, Neurological Bulletin (Nevrologicheskii Vestnik), Folia Psychiatrica et Neurologica Japonica, and Voprosy Sovremennoi Pediatrii (Current Pediatrics). Search terms included: “hopantenic acid” OR “calcium hopantenate” OR “Pantogam” OR “Pantocalcin” OR “homopantothenic acid” OR “N-pantoyl-GABA” combined with “children” OR “paediatric” OR “neurodevelopmental” OR “ADHD” OR “cerebral palsy” OR “epilepsy” OR “cognitive” OR “developmental delay”. The PubMed search strategy was ("hopantenic acid" OR "calcium hopantenate" OR Pantogam OR Pantocalcin OR "homopantothenic acid" OR "N-pantoyl-GABA") AND (child* OR pediatric OR paediatric OR adolescent) AND (neurodevelopmental OR ADHD OR epilepsy OR "cerebral palsy" OR "developmental delay" OR cognitive). No language restrictions were applied. Reference lists of included articles and relevant review papers were hand-searched for additional studies. A prospective review protocol was not registered prior to study initiation.

Data Extraction and Quality Assessment

**Figure 2:**
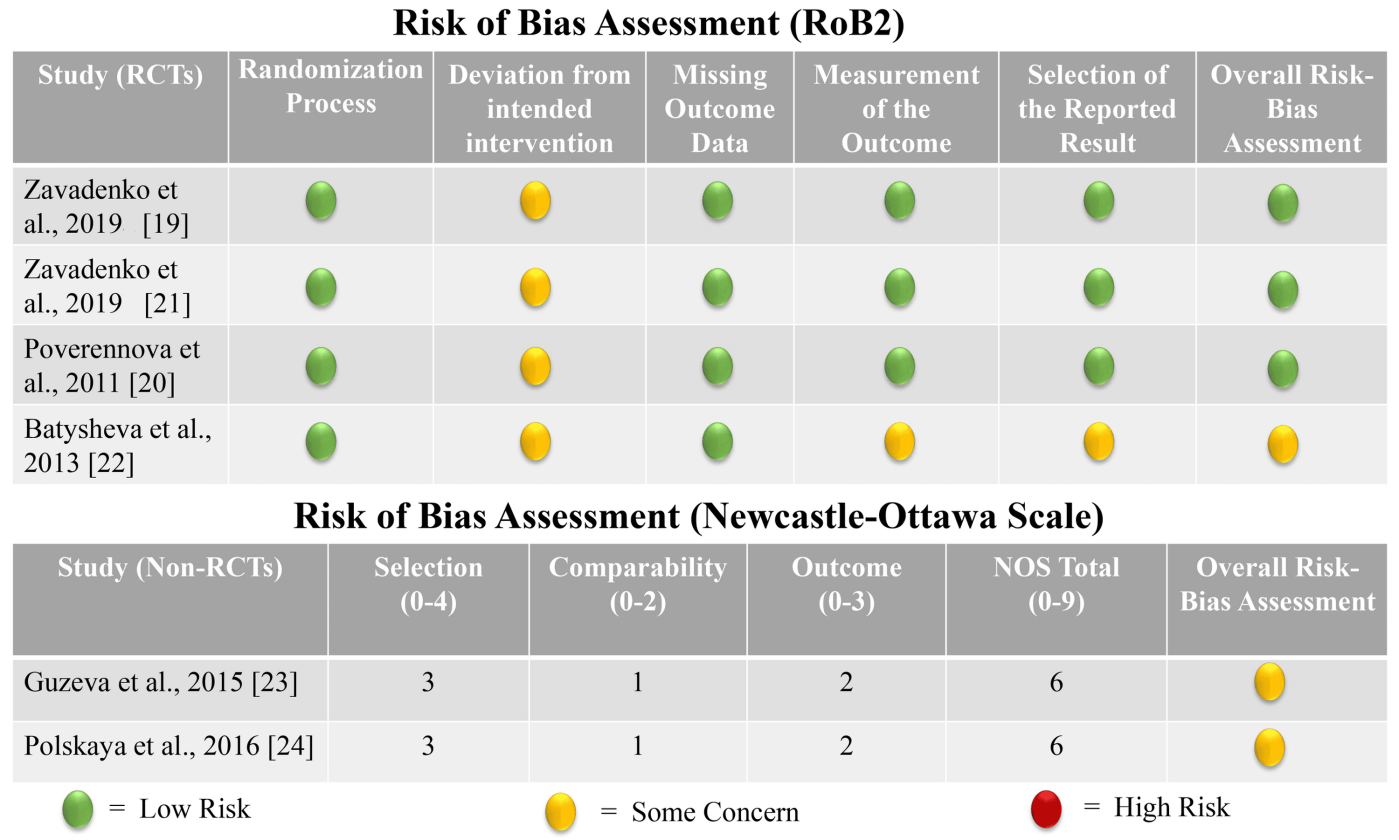
Risk of bias and methodological quality assessment of included studies. First table presents the Cochrane risk of bias 2 (RoB 2) assessment for randomized controlled trials. Second table presents methodological quality assessment of non-randomized comparative studies using the Newcastle-Ottawa Scale (NOS). Because RoB 2 and NOS evaluate different methodological constructs, results should not be interpreted as directly comparable measures of study quality.

Data were extracted independently using a standardized proforma capturing: study design, sample size, patient demographics, diagnostic criteria, intervention details (formulation, dose, duration), comparator, primary and secondary outcome measures, key efficacy results (with p-values and effect estimates where reported), and adverse events. Methodological quality was assessed using the Cochrane Risk of Bias 2 (RoB 2) for randomized controlled trials and the Newcastle-Ottawa Scale for non-randomized comparative studies (Figure 2) [17,18].

Among the four randomized controlled trials, most demonstrated a low overall risk of bias, although some concerns were identified in selected domains. The two non-randomized comparative studies achieved moderate methodological quality scores on the Newcastle-Ottawa Scale. Given the methodological differences between randomized and non-randomized study designs, findings from open-label comparative studies should be interpreted with greater caution than those from blinded randomized controlled trials.

Outcome Measures

The primary outcome of interest was clinical improvement. Although the included studies employed different clinical assessment tools, such as the Diagnostic and Statistical Manual of Mental Disorders (DSM)-IV attention deficit hyperactivity disorder (ADHD) rating scale, seizure frequency reduction, and the Griffiths Mental Development Scales, outcomes from studies eligible for meta-analysis were harmonized into a dichotomous variable (responder versus non-responder) to enable pooled analysis. A responder was defined as a participant demonstrating clinically meaningful improvement according to the predefined criteria of each individual study (e.g., ≥25% reduction in ADHD symptom scores, significant reduction in seizure frequency, or improvement beyond a specified threshold on developmental scales).

For studies that did not report outcomes in a format amenable to dichotomization (e.g., continuous cognitive scores, multidomain assessments, or absence of comparable control data), results were synthesized narratively. These studies were included in the systematic review to provide supportive evidence regarding the efficacy of hopantenic acid but were excluded from quantitative meta-analysis due to methodological heterogeneity. This combined approach allowed comprehensive evaluation of available evidence while maintaining methodological rigor in the pooled analysis.

Results

Study Selection and Characteristics

Six controlled clinical trials met the inclusion criteria, enrolling a total of 439 pediatric participants. The studies were published between 2013 and 2019 in peer-reviewed journals. Three studies employed a double-blind, placebo-controlled design [19-21], and three used an open-label comparative design with a defined control group [22-24]. The clinical populations spanned the following six distinct NDD categories: ADHD, hypoxic-ischemic encephalopathy (HIE) with psychomotor delay, cerebral palsy, partial epilepsy, childhood epilepsy with cognitive impairment, and anxiety. Treatment duration ranged from six weeks to four months (Table 1). Notably, the study by Poverennova et al. included both adolescent and adult participants, introducing a degree of indirectness relative to the predominantly pediatric focus of this review [20].

**Table 1:** Characteristics of included studies. *Population included adolescents and adults (20-49 years). Quantitative synthesis was based on 36 analyzed participants (18 intervention and 18 placebo) with extractable responder data. DB-RCT: double-blind randomized controlled trial; DSM-IV: Diagnostic and Statistical Manual of Mental Disorders, Fourth Edition; GMDS-ER: Griffiths Mental Development Scales - Extended Revised; ARP/BHS: affective-respiratory paroxysms/breath-holding spells; ADHD: attention deficit hyperactivity disorder

Studies	Clinical condition	Total participants	Design	Dose (mg/kg/day)	Duration	Primary outcome
Zavadenko et al. (2019) [19]	ADHD	89	DB-RCT, placebo	30	4 months	DSM-IV ADHD Scale
Zavadenko et al. (2019) [21]	HIE/psychomotor delay	87	DB-RCT, multicenter, placebo	30-50	67 days	Griffiths Scale (GMDS-ER)
Poverennova et al. (2011) [20]*	Partial epilepsy	40	DB-RCT, placebo	20-30	8 weeks	Seizure frequency
Batysheva et al. (2013) [22]	Cerebral palsy	100	RCT, comparative	Standard pediatric dose	90 days	Luria memory test
Guzeva et al. (2015) [23]	Epilepsy+cognitive impairment	63	Comparative	20-30	2 months	Neurocognitive battery
Polskaya et al. (2016) [24]	Affective-respiratory paroxysms (breath-holding spells)	60	Comparative	30-35	3 months	Clinical improvement in affective-respiratory paroxysms (ARP/BHS)

Among the six studies included in the systematic review, four studies were eligible for quantitative meta-analysis. These studies were selected based on the availability of extractable dichotomous outcome data (responders versus non-responders) in both the hopantenic acid and control groups. The included studies evaluated clinical improvement using predefined criteria such as reduction in ADHD symptom scores, improvement in psychomotor development, reduction in seizure frequency, or clinical resolution of breath-holding spells, which could be standardized into a binary outcome.

Two studies were excluded from the meta-analysis. The cerebral palsy study by Batysheva et al. was excluded as it reported outcomes based on normalization across multiple cognitive parameters rather than a clearly defined responder endpoint [22]. Similarly, a study on epilepsy by Guzeva et al., assessing cognitive outcomes, was excluded due to the absence of comparable responder data in the control group. These studies were included in the narrative synthesis to provide supporting evidence but were not quantitatively pooled due to methodological heterogeneity [23].

Attention Deficit Hyperactivity Disorder

Zavadenko et al. conducted a prospective, multicenter, double-blind, placebo-controlled trial in 100 children aged 6-12 years with ADHD. Patients received hopantenic acid (Pantogam) at 30 mg/kg/day or placebo for four months, with 89 participants completing the study. Clinical response rates (>25% reduction in DSM-IV ADHD score) were higher in the Pantogam group at months three and four compared with placebo. Pantogam also produced a significant reduction in Clinical Global Impression-Severity (CGI-S) disease severity scores and significantly improved sustained attention on the Toulouse-Piéron test (p<0.05). Functional impairment scores on the Weiss Functional Impairment Rating Scale-Parent Report (WFIRS-P) improved across family, school, self-concept, and risky activity domains. The treatment was well tolerated, with adverse events comparable to placebo (Table 2) [19].

**Table 2:** Summary of primary efficacy outcomes across included studies. Comparative summary of clinical studies evaluating the efficacy of hopantenic acid in pediatric neurological and neurodevelopmental conditions, including ADHD, hypoxic-ischemic encephalopathy (HIE), epilepsy, cerebral palsy, cognitive impairment, and anxiety-related disorders. The table presents study populations, outcome measures, treatment responses in hopantenic acid versus control groups, and statistical significance. Across studies, hopantenic acid demonstrated improvements in behavioral symptoms, cognitive performance, seizure reduction, memory function, and clinical symptom severity compared with controls. ADHD outcomes were assessed using DSM-IV criteria, while neurodevelopmental and neurocognitive improvements were evaluated using standardized clinical scales and cognitive batteries. Statistical significance was observed in most studies, supporting the potential therapeutic role of hopantenic acid in pediatric neurodevelopmental disorders. CI: cognitive impairment; BHS: breath-holding spells; DSM-IV: Diagnostic and Statistical Manual of Mental Disorders, Fourth Edition; ADHD: attention deficit hyperactivity disorder; CGI-S: Clinical Global Impression-Severity

Studies	Condition	Outcome measure	Hopantenic acid group	Control group	p-Value/significance
Zavadenko et al. (2019) [19]	ADHD (n=89)	DSM-IV ADHD Response (>25% reduction)	68.9% responders at 4 months	61.4% responders	Responder difference shows a trend with hopantenic acid group; CGI-S improved (p<0.05)
Zavadenko et al. (2019) [21]	HIE (n=87)	Griffiths Scale >6% improvement	63.6% improved	39.5% improved	0.021
Poverennova et al. (2011) [20]	Partial epilepsy (analyzed n=36)	Seizure frequency reduction	Mean seizure frequency reduction: 72.1%	Mean seizure frequency reduction: 16.7%	<0.05
Batysheva et al. (2013) [22]	Cerebral palsy (n=100)	Luria test normalization (day 90)	19.05% normalized	2.04% normalized	0.031
Guzeva et al. (2015) [23]	Epilepsy+CI (n=63)	Neurocognitive battery	Significant reduction in cognitive impairment; EEG normalization observed in 15% of patients	No significant cognitive improvement	<0.05
Polskaya et al. (2016) [24]	Affective-respiratory paroxysms (breath-holding spells) (n=60)	Clinical improvement rate	73.3% improved	16.7% improved	<0.05

Psychomotor Developmental Delay in Premature Infants With Hypoxic-Ischemic Encephalopathy

Zavadenko et al. conducted a double-blind, multicenter, placebo-controlled study in 87 premature infants aged six to 12 months with psychomotor developmental delay following hypoxic-ischemic CNS injury. Infants received standard therapy plus hopantenic acid (Pantogam) syrup (30-50 mg/kg/day) or placebo for 67 days. Improvement in psychomotor development (>6% increase in Griffiths Mental Development Scales - Extended Revised {GMDS-ER} total score) was observed in 63.6% of the Pantogam group compared with 39.5% of the placebo group (p=0.021). Significant benefits were noted in personal-social functioning and object manipulation, with additional trends toward improvement in motor activity, hearing and speech, and visual-motor coordination. Pantogam showed greater benefit in late preterm infants and was well tolerated, with a safety profile comparable to placebo (Table 2) [21].

Cerebral Palsy With Cognitive Impairment

Batysheva et al. conducted a randomized controlled trial in 100 children aged eight to 14 years with cerebral palsy and cognitive impairment [22]. A total of 50 patients received hopantenic acid (Pantocalcin) plus standard therapy, while 50 received standard therapy alone for 90 days. By day 90, normal memory recall scores on the Luria test were achieved in 19.05% of the Pantocalcin group compared with 2.04% of controls (p=0.031). Significant improvements were also observed in visual memory and concentration capacity on the Toulouse-Piéron test, along with reduced anxiety levels. However, visual-motor skills did not differ significantly between groups. Pantocalcin was well tolerated, with no treatment-related serious adverse events reported (Table 2) [22].

Epilepsy With Cognitive Impairment in Children

Two studies assessed hopantenic acid as adjunctive therapy in pediatric epilepsy. Guzeva et al. studied 63 children aged four to seven years with cryptogenic or symptomatic epilepsy and comorbid cognitive impairment [23]. A total of 40 children received Pantogam (20-30 mg/kg/day) as add-on to antiepileptic therapy; 23 served as comparators without Pantogam. Assessment using the Lüscher color test, “playing the piano” test, and the “three” and “fourth is extra” tests demonstrated significant reduction in cognitive impairment in the Pantogam group relative to comparators. EEG normalization was observed in 15% of patients at two months. Importantly, no increase in seizure frequency was observed, confirming the anticonvulsant safety profile of hopantenic acid in epilepsy (Table 2) [23].

Poverennova et al. conducted a randomized, double-blind, placebo-controlled trial of Pantogam Active (D,L-hopantenic acid) in 40 patients with partial epilepsy (20 Pantogam Active, 20 placebo) at a dose of 1200-1800 mg/day for eight weeks [20]. Notably, the study included adolescents and adults (20-49 years), introducing a degree of indirectness relative to the predominantly pediatric populations represented in the remaining studies. Nevertheless, the findings were considered relevant to the review objectives because they provided controlled clinical evidence in a neurological condition associated with cognitive, behavioral, and developmental outcomes. Seizure frequency decreased by 72.1% in the Pantogam group versus 16.7% with placebo. Significant improvements in long-term memory and anxiety (Hospital Anxiety and Depression Scale) were also observed, together with improvements in quality of life as assessed by the Quality of Life in Epilepsy Inventory-31 (QOLIE-31) scale [20]. For quantitative synthesis, only participants with extractable dichotomous responder outcome data were included (18 participants in the Pantogam group and 18 participants in the placebo group), resulting in a total analyzable sample of 36 participants for the meta-analysis (Table 2).

Anxiety

Polskaya et al. evaluated Pantogam syrup 10% (100 mg/mL, 30-35 mg/kg/day for three months) in 60 children aged two to four years with anxiety [24]. A total of 30 children received Pantogam; 30 received psychological correction only. Clinical improvement was observed in 73.3% of patients in the Pantogam group compared with 16.7% in the comparison group. Anxiety levels, assessed using standardized instruments, decreased significantly following neuroprotective therapy. EEG analysis revealed a significant reduction in slow-wave rhythm power (p<0.05), indicating improved functional maturation of the brain. These results suggest that hopantenic acid addresses both the clinical manifestations and the underlying neurophysiological substrate of breath-holding spells (Table 2) [24].

Safety Profile Across Studies

Overall, across all six included trials, hopantenic acid demonstrated acceptable short-term tolerability in the included clinical trials. However, given the modest sample sizes, relatively short follow-up durations, and limited number of studies, the available evidence is insufficient to fully characterize uncommon or long-term adverse events. Consequently, larger studies with extended follow-up are needed to establish its long-term safety profile. In the three double-blind placebo-controlled trials, the incidence and spectrum of adverse events in the hopantenic acid groups were comparable to those in the placebo groups [19-21]. No treatment-related serious adverse events were reported in any pediatric trial. The most commonly reported adverse effects were mild and transient, including occasional rhinitis, drowsiness, and hyperexcitability, which resolved without dose modification. Notably, in pediatric epilepsy populations, hopantenic acid did not increase seizure frequency, a critical safety consideration for nootropic agents used in epilepsy [22-24]. The safety data are consistent with the pharmacological profile of a partial GABA-B agonist, which by definition avoids the sedation and tolerance associated with full agonism [25].

Meta-analysis findings

Four studies comprising a total of 272 participants (hopantenic acid: n=137; control: n=135) were included in the quantitative synthesis [19-21,24]. Using a random-effects Mantel-Haenszel model, the pooled analysis demonstrated a statistically significant improvement in clinical outcomes with hopantenic acid compared to control (risk ratio {RR}=2.13, 95% confidence interval {CI}: 1.11-4.08; p=0.02).

Subgroup analyses indicated variability in treatment effects across clinical conditions. In the ADHD study, the proportion of responders was higher in the hopantenic acid group (31/45) compared with control (27/44); however, this difference was not statistically significant (RR=1.12, 95% CI: 0.83-1.52; p=0.46). In contrast, statistically significant effects were observed in partial epilepsy (RR=4.33, 95% CI: 1.48-12.66; p=0.007), anxiety (RR=4.40, 95% CI: 1.92-10.08; p=0.0005), and hypoxic-ischemic encephalopathy (RR=1.61, 95% CI: 1.05-2.48; p=0.03).

Substantial heterogeneity was observed across studies (I²=81%), likely due to differences in clinical conditions, outcome measures, and study designs. A random-effects model was applied to account for this variability, and subgroup analyses suggested condition-specific differences in treatment effects. Despite the heterogeneity, the overall effect remained significant; however, results should be interpreted with caution. Furthermore, subgroup differences were statistically significant (χ²=13.78, df=3, p=0.003; I²=78.2%), indicating that treatment effects varied across disease categories.

**Figure 3:**
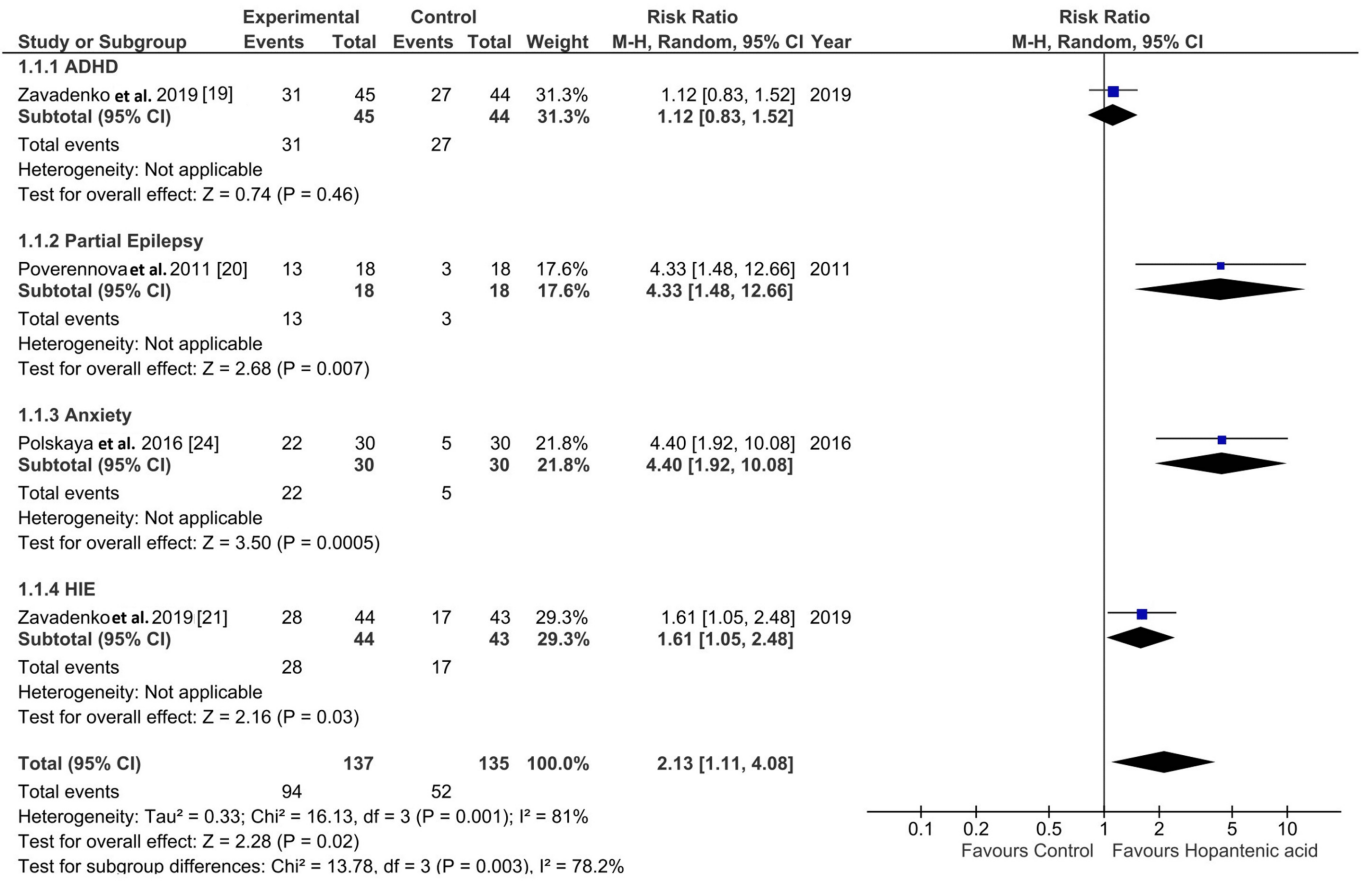
Forest plot of clinical efficacy of hopantenic acid across NDDs and neurological disorder. Clinical improvement was defined according to each individual study as follows: ADHD (>25% reduction in DSM-IV score), HIE (>6% improvement on the GMDS-ER), partial epilepsy (reduction in seizure frequency), and affective-respiratory paroxysms (overall clinical improvement). Risk ratios (RR) >1 favor hopantenic acid because the event represents clinical improvement. The study by Poverennova et al. included adolescents and adults (20-49 years), introducing a degree of indirectness relative to the predominantly pediatric review population. Because the included studies evaluated clinically heterogeneous conditions and outcome definitions, the pooled estimate should be interpreted as an exploratory quantitative synthesis [20]. Substantial heterogeneity was observed (I²=81%); therefore, the overall pooled estimate should be interpreted with caution. NDDs: neurodevelopmental disorders; DSM-IV: Diagnostic and Statistical Manual of Mental Disorders, Fourth Edition; GMDS-ER: Griffiths Mental Development Scales - Extended Revised; ADHD: attention deficit hyperactivity disorder; HIE: hypoxic-ischemic encephalopathy

As the outcome of interest represented clinical improvement, a beneficial event, an RR greater than 1 indicates a favorable effect of hopantenic acid. Accordingly, the pooled estimate suggests that treatment with hopantenic acid is associated with a significantly increased likelihood of clinical improvement compared with control. Overall, these findings suggest a positive therapeutic effect of hopantenic acid across multiple neurodevelopmental and neurological conditions, with the most pronounced effects observed in epilepsy and anxiety (Figure 3).

Effects of hopantenic acid on neurodevelopmental milestones

Evidence from multiple randomized and clinical studies suggests that hopantenic acid exerts beneficial effects across major neurodevelopmental domains collectively represented by language, cognition, motor coordination, and social communication (LCMS). Its neuroprotective and neurometabolic actions are primarily attributed to modulation of GABAergic neurotransmission, enhancement of neuronal metabolism, improvement in cerebral bioenergetics, and increased resistance of neural tissue to hypoxia and metabolic stress.

In children with hypoxic-ischemic encephalopathy (HIE), treatment with hopantenic acid demonstrated clinically meaningful improvements in psychomotor development, including gains in hearing and speech, locomotor activity, eye-hand coordination, and personal-social functioning. In ADHD, therapy was associated with improved attention maintenance, reduced functional impairment, and better school and behavioral performance. Studies involving children with epilepsy and cognitive impairment reported improvements in memory, learning ability, neurocognitive performance, and EEG normalization without increasing seizure frequency, indicating a favorable neurocognitive safety profile. Furthermore, in cerebral palsy, hopantenic acid showed positive effects on visual memory, concentration, mental activity, fatigue, and emotional stability, supporting its role in cognitive rehabilitation. Additional studies in anxiety-related pediatric conditions demonstrated reductions in anxiety levels and behavioral dysregulation, indirectly contributing to improved social interaction and communication skills. Collectively, these findings indicate that hopantenic acid may support multidimensional neurodevelopment by improving cognitive processing, language-related functions, motor integration, and sociobehavioral outcomes across a range of pediatric neurological and neurodevelopmental disorders (Table 3).

**Table 3:** Narrative mapping of reported clinical outcomes across the author-derived LCMS domains. LCMS: language, cognition, motor coordination, and social communication; HIE: hypoxic-ischemic encephalopathy; CI: cognitive impairment; ADHD: attention deficit hyperactivity disorder

Neurodevelopmental milestone	Studies	Evidence
Language	Zavadenko et al. (2019) [21] - HIE	Improvement seen in “hearing and speech” domain on Griffiths Scale
	Guzeva et al. (2015) [23] - epilepsy+cognitive Impairment	Improvement in neurocognitive tests, learning, and cognitive processing
Cognition	Batysheva et al. (2013) [22]- cerebral palsy	Improved visual memory, attention, concentration, activity, and fatigue
	Guzeva et al. (2015) [23] - epilepsy+CI	Significant reduction in cognitive impairment
	Poverennova et al. (2011) [20] - partial epilepsy	Improved long-term memory and quality of life
	Zavadenko et al. (2019) [19] - ADHD	Better attention maintenance, school learning, self-concept
Motor coordination	Zavadenko et al. (2019) [21] - HIE	Improvement in “eye and hand coordination” and “locomotor” domains
	Batysheva et al. (2013) [22] - cerebral palsy	Evaluated visual-motor skills; limited/no major effect specifically stated
Social communication	Zavadenko et al. (2019) [21] - HIE	Improvement in “personal-social” domain
	Zavadenko et al. (2019) [19] - ADHD	Improved family interaction, school learning, risky activities, self-concept
	Polskaya et al. (2016) [24] - anxiety	Reduced anxiety and behavioral improvement supporting social interaction

Discussion

This systematic review consolidates the clinical trial evidence for hopantenic acid in pediatric neurodevelopmental disorders and, to our knowledge, represents the first systematic review and meta-analytic synthesis of this body of evidence. Across six controlled trials encompassing 439 children with six distinct NDD subtypes, hopantenic acid consistently demonstrated improvements in cognitive function, psychomotor development, attention, memory, and behavioral outcomes, with a safety profile comparable to placebo.

Meta-Analytic Interpretation

The quantitative synthesis demonstrated a significant overall benefit of hopantenic acid (RR=2.13, 95% CI: 1.11-4.08), indicating that treated patients were more than twice as likely to achieve clinical improvement compared to controls. However, subgroup analysis revealed variability in treatment effects, with strong efficacy observed in epilepsy and anxiety, moderate effects in hypoxic-ischemic encephalopathy, and no statistically significant benefit in ADHD. This variability likely reflects differences in underlying pathophysiology and outcome definitions across conditions. The high heterogeneity (I²=81%) further supports the presence of clinical and methodological diversity among studies, reinforcing the importance of interpreting pooled results within the context of subgroup findings.

Interpretation of these findings should also consider the methodological quality of the included studies. Among the randomized controlled trials, most demonstrated a low overall risk of bias, although some concerns were identified in selected domains. The two non-randomized comparative studies achieved moderate methodological quality according to the Newcastle-Ottawa Scale. Given the methodological differences between randomized and non-randomized study designs, findings from the open-label comparative studies should be interpreted with greater caution than those from blinded randomized controlled trials, thereby reducing the overall certainty of the evidence.

Mechanistic Rationale

The clinical benefits observed across diverse neurodevelopmental disorders are consistent with the multimodal pharmacological profile of hopantenic acid. As a selective presynaptic partial GABA-B agonist, it modulates long-term potentiation (LTP) by selectively binding to the R1 subunit without fully activating the R2 subunit, thereby preserving the excitation-inhibition balance necessary for synaptic plasticity while avoiding excessive sedation and tolerance [15,26]. Supporting this mechanism, Davies et al. demonstrated that GABA-B receptor blockade prevents LTP induction, confirming the importance of GABA-B signaling in normal synaptic plasticity [27].

In addition, hopantenic acid enhances neuronal metabolism by improving glucose utilization, nucleic acid metabolism, and adenosine triphosphate (ATP) synthesis [15]. It also stimulates acetylcholine synthesis in the cerebral cortex and hippocampus, contributing to improvements in attention and memory [11]. Its neuroprotective effects against hypoxia and toxic injury are particularly relevant in hypoxic-ischemic encephalopathy, where neuronal vulnerability is prominent [28]. Together, these mechanisms provide a plausible biological basis for the broad neurocognitive benefits observed across the NDD spectrum.

LCMS-Associated Neurodevelopmental Benefits

Importantly, the observed benefits of hopantenic acid were not limited to isolated symptom reduction but extended across multiple neurodevelopmental domains represented within the LCMS framework, including language, cognition, motor coordination, and social communication. Improvements in hearing and speech development, attention maintenance, visual memory, psychomotor performance, behavioral regulation, and adaptive social functioning suggest that hopantenic acid may exert a multidimensional effect on neurodevelopmental maturation. These findings are clinically relevant because functional outcomes in pediatric neurodevelopmental disorders are inherently multidomain in nature and are often poorly captured by single-symptom assessment tools [29]. The broad LCMS-associated improvements observed across studies support the hypothesis that modulation of synaptic plasticity and neuronal metabolism by hopantenic acid may contribute to more integrated neurodevelopmental benefits rather than isolated symptomatic control alone. Although these findings are encouraging, it should be noted that the evidence of developmental improvement in hypoxic-ischemic encephalopathy is based on a single placebo-controlled study with a relatively short follow-up period. Therefore, these observations should be considered preliminary and require confirmation in larger studies with longer-term assessment.

Comparison With Existing Therapies

For ADHD, the current first-line treatment in most international guidelines is methylphenidate, which acts primarily through catecholaminergic (dopamine/norepinephrine) mechanisms. While methylphenidate demonstrates robust effect sizes in controlled trials, its use is associated with appetite suppression, insomnia, growth attenuation, and cardiovascular effects [30]. Moreover, regulatory approval and access to methylphenidate vary across countries, resulting in differences in treatment availability and prescribing practices. Hopantenic acid offers a mechanistically distinct approach targeting the GABAergic rather than catecholaminergic system, with a notably benign side-effect profile. The ADHD trial data suggest moderate efficacy, with response rates approaching those of placebo but with significant improvements in functional domains and sustained attention [31]. These observations should be interpreted as contextual rather than practice-changing, as no direct head-to-head comparisons with established therapies are currently available. Consequently, while hopantenic acid may represent a potential therapeutic option in selected clinical settings, its comparative efficacy and role as an alternative or adjunctive therapy require confirmation in well-designed comparative clinical trials.

For cerebral palsy, the therapeutic landscape for cognitive impairment is particularly barren. Few pharmacological interventions have demonstrated efficacy for the cognitive dimensions of CP, and most management strategies focus on motor rehabilitation [32]. The significant improvement in long-term memory demonstrated by Batysheva et al. represents a clinically meaningful finding that warrants further investigation [22].

In pediatric epilepsy, the use of nootropic agents is constrained by the theoretical risk of seizure exacerbation. The data from Poverennova et al. and Guzeva et al. are reassuring, demonstrating not only cognitive improvement but also seizure reduction rather than exacerbation [20,23]. This is consistent with the known anticonvulsant properties of hopantenic acid mediated through GABAergic enhancement [15].

Limitations

This review has several limitations. The available evidence is based on a small number of studies with modest sample sizes, short follow-up durations, and substantial clinical and methodological heterogeneity in study populations, intervention formulations, comparator groups, and outcome measures. One included study enrolled adolescents and adults, introducing a degree of indirectness relative to the predominantly pediatric focus of this review. The pooled meta-analysis should therefore be interpreted as an exploratory synthesis rather than definitive evidence of efficacy across all neurodevelopmental disorders. In addition, the LCMS framework represents an author-derived interpretive framework and should not be considered a validated clinical assessment model. Publication bias and formal Grading of Recommendations Assessment, Development and Evaluation (GRADE) certainty-of-evidence assessments were not performed because of the limited number of eligible studies. Consequently, larger, multicenter, condition-specific randomized controlled trials using standardized outcome measures are needed to confirm the efficacy and long-term safety of hopantenic acid.

## Conclusions

This systematic review and meta-analysis suggest that hopantenic acid may provide short-term clinical benefits in selected pediatric neurodevelopmental and neurological conditions, with acceptable short-term tolerability in the included studies. The pooled quantitative synthesis indicated an increased likelihood of clinical improvement compared with control; however, treatment effects varied substantially across clinical conditions, with more pronounced effects observed in epilepsy- and anxiety-related disorders, moderate benefits in hypoxic-ischemic encephalopathy, and comparatively modest or non-significant effects in ADHD. Given the substantial clinical heterogeneity, differing outcome definitions, and variability in study designs, the pooled findings should be interpreted as an exploratory synthesis rather than definitive evidence of uniform efficacy across the spectrum of neurodevelopmental disorders. Narrative synthesis further suggested improvements across multiple LCMS-associated domains, including language, cognition, motor coordination, and social communication. However, the LCMS framework represents an author-derived interpretive framework developed to organize multidimensional outcomes reported across the included studies and should not be interpreted as a validated clinical assessment model or as confirmation of multidomain neurodevelopmental efficacy. Similarly, while the pharmacological properties of hopantenic acid provide a plausible biological rationale for the observed findings, mechanistic plausibility should be regarded as hypothesis-generating rather than confirmatory evidence of clinical benefit.

Interpretation of these findings is limited by substantial inter-study heterogeneity, non-equivalent outcome measures, modest sample sizes, mixed study designs, short follow-up durations, and the geographic concentration of evidence, with most studies originating from the Russian Federation. In addition, one included study enrolled adolescents and adults, introducing a degree of indirectness relative to the predominantly pediatric focus of this review. Accordingly, although hopantenic acid appears to be a promising therapeutic candidate for selected pediatric neurodevelopmental and neurological conditions, its clinical utility requires confirmation through rigorously designed, adequately powered, multicenter randomized controlled trials in well-defined pediatric populations using standardized and internationally validated outcome measures. Future investigations should also evaluate long-term safety, optimized pediatric dosing strategies, condition-specific efficacy, head-to-head comparisons with existing therapies, and neuroimaging- or biomarker-based mechanistic studies to better define the therapeutic role of hopantenic acid.
